# Photobodies: Light‐Activatable Single‐Domain Antibody Fragments

**DOI:** 10.1002/anie.201912286

**Published:** 2019-12-12

**Authors:** Benedikt Jedlitzke, Zahide Yilmaz, Wolfgang Dörner, Henning D. Mootz

**Affiliations:** ^1^ Institute of Biochemistry Department of Chemistry and Pharmacy University of Muenster Wilhelm-Klemm-Str. 2 48149 Münster Germany

**Keywords:** antibodies, nanobodies, photochemistry, protein–protein interactions, unnatural amino acids

## Abstract

Photocaged antibody fragments, termed photobodies, have been developed that are impaired in their antigen‐binding capacity and can be activated by irradiation with UV light (365 nm). This rational design concept builds on the selective photocaging of a single tyrosine in a nanobody (a single‐domain antibody fragment). Tyrosine is a frequently occurring residue in central positions of the paratope region. *o*‐Nitrobenzyl‐protected tyrosine variants were incorporated into four nanobodies, including examples directed against EGFR and HER2, and photodeprotection restores the native sequence. An anti‐GFP photobody exhibited an at least 10 000‐fold impaired binding affinity before photodeprotection compared with the parent nanobody. A bispecific nanobody–photobody fusion protein was generated to trigger protein heterodimerization by light. Photoactivatable antibodies are expected to become versatile protein reagents and to enable novel approaches in diagnostic and therapeutic applications.

## Graphical abstract

**Lights on, action! Ready, steady, bind**! Nanobodies are exquisite tools in basic research, diagnostics, and therapy. Photobodies are photoactivatable variants of these single‐domain antibody fragments, designed with a single photo‐caging group in the paratope region. Photobodies, in monovalent and multivalent forms, are expected to be versatile protein reagents for the temporal and spatial control of antibody binding.

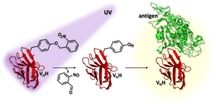

Antibodies recognize their antigens with high affinity and selectivity. They are indispensable protein reagents in basic research and biomedicine, for example to detect and enrich their binding partners and for diagnostic and therapeutic applications. Bispecific antibodies contain two different paratope sites for antigen binding and trigger the heterodimerization of their respective epitopes.^1^ Antibody binding is mediated through extended complementary interaction surfaces with the epitope. The three complementarity‐determining regions (CDRs) in the variable domains of the immunoglobulin scaffold represent the hot spots of sequence diversity and structural malleability to provide the most important contributions to binding.

We envisaged the rational design of novel protein reagents in which the binding of the antibody variable domain(s) to its cognate epitope is rendered light dependent. Photoactivatable and photoswitchable molecules are exquisite tools to study biological systems with high temporal and spatial resolution,^2^ to develop new biomaterials,^3^ and they have great potential for therapeutic purposes in the field of photopharmacology.^4^ Previous examples of light‐controlled molecules covered small molecules and ligands, enzymes, ion channels, various proteins, nucleic acids, lipids, and others.^5^ However, the design of light‐dependent antibodies, with a direct and defined activation of the antibody–antigen interaction, has not been reported yet.^6^ Previous efforts to modulate this binding event in a light‐dependent manner include chemical photocaging of the antigen,^7^ global, unspecific and uncharacterized coating of the antibody with up to 50 chemical photocaging groups per protein,^8^ and photoinduced cleavage of synthetically fused epitopes that act as a non‐covalent, bivalent inhibitor.^9^

We focused on single‐domain antibody fragments, also referred to as nanobodies, which are the variable domains of heavy‐chain‐only antibodies of camelids (V_H_H).^10^ Nanobodies bind their epitope monovalently and typically with affinities in the low nanomolar or sometimes even picomolar range. They are highly stable in monomeric form and can be efficiently expressed in *Escherichia coli*. With these and other favorable properties, nanobodies recently have gained considerable attention. They are being explored for a large variety of applications, mostly in basic research and diagnostics, but they are also considered attractive in therapy.10a, 11

Our concept to rationally design light‐activatable nanobodies, termed photobodies, is based on the idea of structurally perturbing and thereby impairing the nanobody–antigen interaction by the introduction of a sterically demanding photocaging group at a central and neuralgic position in the paratope region of the nanobody. Light‐induced cleavage of the cage group would furnish the native nanobody sequence to reestablish binding (Scheme 1). We observed that the CDR loops of nanobodies show a high frequency of tyrosine residues. The same holds true for several additional residues on the nanobody core domain that are often implicated in antigen binding.^12^ We further inspected structures of nanobody–antigen complexes deposited in the Protein Data Bank and found that these tyrosines are often part of the nanobody–antigen interaction interface. Therefore, we selected tyrosine side chains for our photocaging approach.

**Scheme 1:**
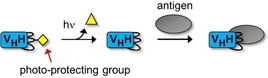
Principle of a light‐activatable nanobody, termed photobody.

As the first nanobody test case, we selected an anti‐GFP nanobody (GFP‐enhancer; PDB: 3K1K).^13^ The structure with its antigen reveals a Tyr residue (Y37) located in the center of the extended protein–protein interaction interface (Figure 1 A,B). The side chain of Y37 is in nearly perpendicular orientation to the β‐barrel structure of GFP. Remarkably, the Y37 side chain is almost completely engulfed by other residues of the binding interface in the wild‐type nanobody–GFP complex. We expected that a photolabile protecting group would not only disrupt hydrogen bonding of the hydroxyl group, but also prevent the formation of multiple interactions in the vicinity of Y37 through its steric demand and therefore have a dramatic effect on the binding affinity.

**Figure 1:**
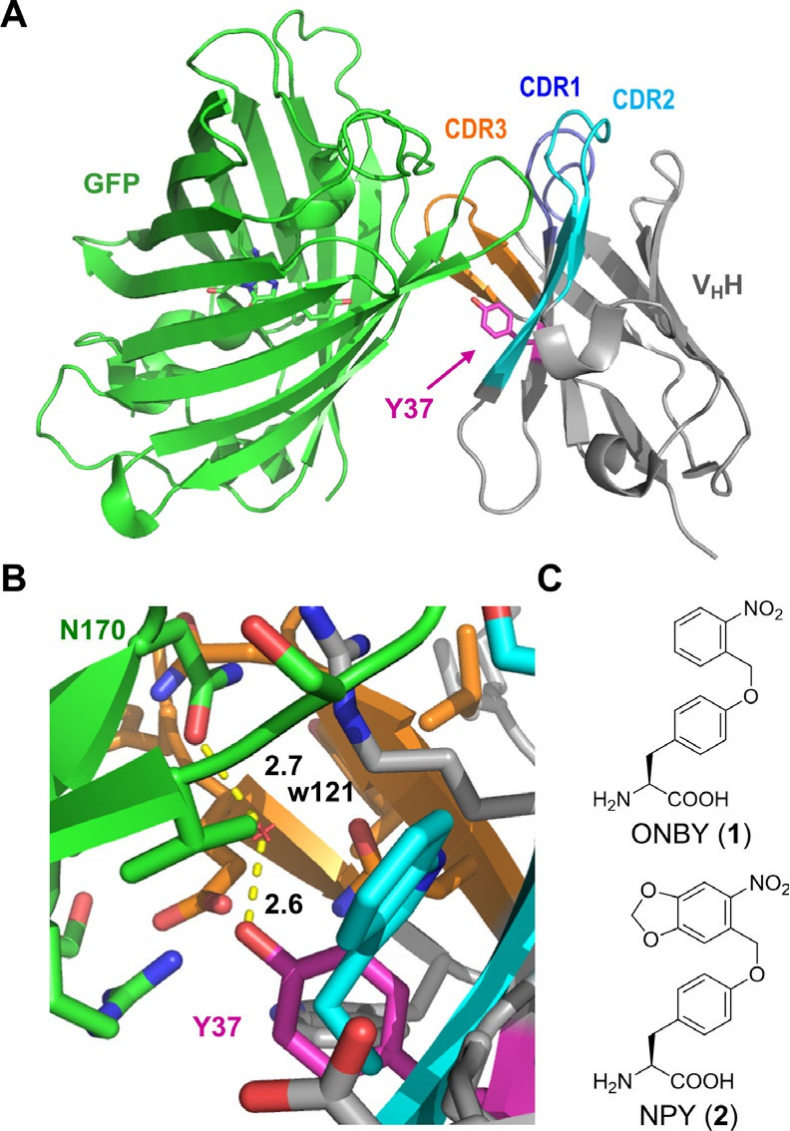
Rational design of an anti‐GFP photobody. A key tyrosine residue is selected for replacement with a photocaged tyrosine residue. A) Crystal structure of an anti‐GFP nanobody with its antigen (PDB: 3K1K).^13^ B) Close‐up of the surroundings of Y37 in the nanobody–antigen interaction interface. C) Chemical structures of photocaged tyrosine variants.

For photocaged Tyr variants, we turned our attention to *o*‐nitrobenzyl‐protected Tyr (ONBY, compound **1**) and its methylenedioxy‐derivative nitropiperonyl tyrosine (NPY, **2**; Figure 1 C). The genetic incorporation of **1** into proteins by the amber stop codon suppression technology has been previously reported with a *Methanococcus jannaschii* anti‐tyrosyl tRNA synthetase (TyrRS, *Mj*TyrRS) mutant selected for ONBY and its cognate *Mj*tRNA for expression in *E. coli*.^14^ Both **1** and **2** have been encoded in mammalian cells using a mutant of pyrrolysine‐tRNA synthetase.^15^ In contrast, to our knowledge, the incorporation of **2** has not yet been shown in *E. coli* using the MjTyrRS system.15a, 15b

To test our concept, we produced both the wild‐type anti‐GFP nanobody (construct **3**) and its two caged photobody variants with Y37ONBY (**4**) and Y37NPY (**5**). For the latter two, the orthogonal pair of the *Mj*TyrRS and *Mj*tRNA for incorporation of **1** 14 was co‐expressed in the *E. coli* production host. Amino acids **1** or **2** were added to the growth medium at 1 mm concentration. NPY (**2**) was found to be accepted as a *Mj*TyrRS(ONBY) substrate, albeit with slightly reduced expression levels. Figure 2 A shows the purified nanobodies. Consistent with our previous findings on periplasmic expression,^16^ mass spectrometry (MS) analysis of the purified protein fractions suggested the presence of only negligible contents of the reduced *o*‐aminobenzyl forms (OABY and APY; ≤5 % and ≤3 %, respectively; Supporting Information, Figures S1 A,B and S2). No prematurely deprotected photobody with the mass of the wild‐type nanobody could be detected. Protein stabilities showed very similar melting temperatures for the wild‐type nanobody and the ONBY–photobody (Supporting Information, Figure S1 C), consistent with the idea that photocaging of a surface‐exposed tyrosine residue has a negligible impact on protein folding. Photodeprotection (*λ*=365 nm) occurred virtually quantitatively and within a few seconds (Figure 2 C,D and Supporting Information, Figure S2).

**Figure 2:**
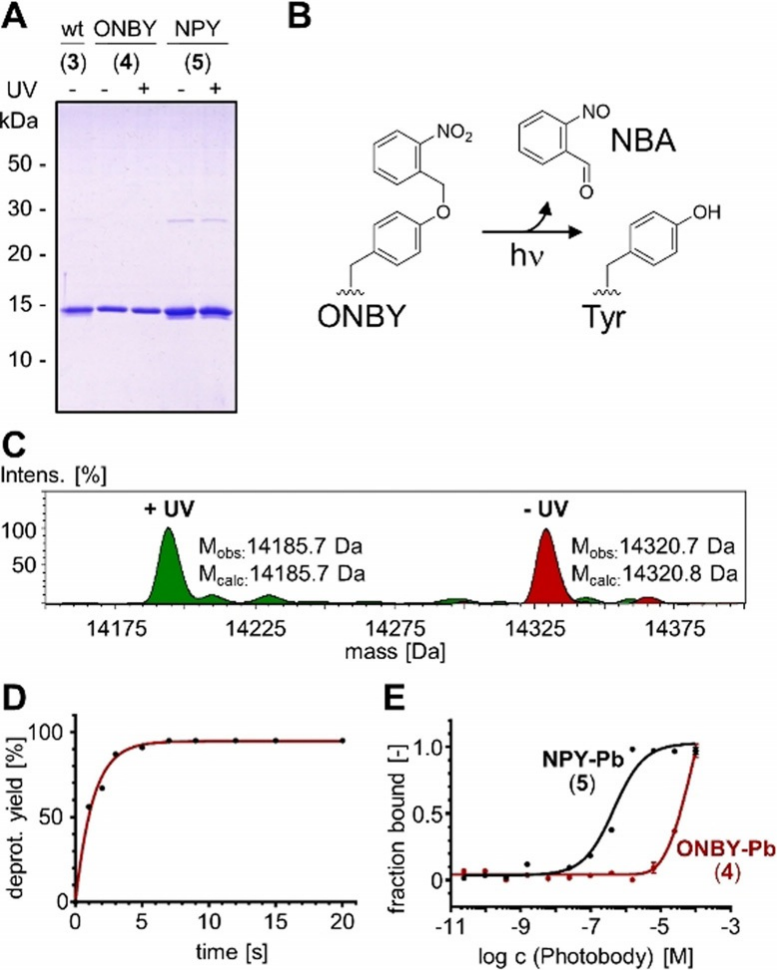
Characterization of the anti‐GFP photobodies **4** and **5**. A) Coomassie‐stained SDS‐PAGE gel showing purified proteins before (‐) and after (+) exposure to UV light. B) Photodeprotection reaction of ONBY (**1**). NBA=nitrosobenzaldehyde. C) ESI‐MS analysis of the ONBY‐photobody (**4**) before (red) and after (green) photodeprotection with *λ*=365 nm. D) Time‐course of photodeprotection of the ONBY–photobody (**4**) determined by ESI‐MS analysis. E) Determination of binding affinities of the caged photobodies **4** and **5** for sfGFP determined by microscale thermophoresis (MST). See Figure S2 in the Supporting Information for additional data on the NPY–photobody **5**.

We next addressed the crucial question on the intended loss of binding affinity of the photobodies and the re‐activation by light. We performed microscale thermophoresis (MST) experiments with superfolder GFP (sfGFP) as the fluorescent antigen and added the photobody in a dilution series. To our delight, we could not reach saturation in binding for the ONBY–photobody (**4**), indicating an estimated dissociation constant *K*_d_ of ≥10 μm (Figure 2 E and Supporting Information, Figure S3 A). This is at least an approximately 10 000‐fold impairment compared to the binding constant reported for the wild‐type nanobody (**1**; *K*_d_=1.1 nm for sfGFP^17^ and 0.6 nm for GFP^13^). Unexpectedly, the NPY‐caged photobody (**5**) was less impaired (approximately 420‐fold), with a *K*_d_ of 0.46±0.02 μm (Supporting Information, Figure S3 B). A possible explanation for this finding is that other binding contributions from the NPY protecting group have accidentally and partially compensated for the impairment caused by the steric effect in this particular case. We therefore abandoned the NPY variant in the subsequent study and focused on the ONBY–photobody (**4**), although the sterically more demanding NPY is likely to be useful or even superior in other cases. To assay the photodeprotected photobodies for reconstituted antigen‐binding capacity, we realized the limited sensitivity of our MST assay to measure with sfGFP concentrations below 10 nm, which is 10‐fold higher than the expected binding affinity. Nevertheless, the assay sufficed to show that the ONBY–photobody (**4**) regained binding affinity after photodeprotection (Supporting Information, Figure S3 A).

To accurately measure the binding affinities, we displayed the nanobodies on the surface of *E. coli* cells using the AIDA autodisplay system^18^ and measured antigen binding (sfGFP) by flow cytometry (Figure 3 and Supporting Information, Figure S4). *E. coli* cells presenting the wild‐type anti‐GFP nanobody bound sfGFP with a *K*_d_ of 3.0 nm, suggesting the nanobody was displayed in fully functional form. To display the ONBY–photobody, we combined the AIDA autodisplay with the amber stop codon suppression technique.^19^ Prior to irradiation of the presenting cells, no sfGFP binding could be detected for concentrations up to about 10 μm, consistent with the results from our MST assay. We then added sfGFP to cells that had been irradiated for 45 s (*λ*=365 nm) and could determine a *K*_d_ of 0.90±0.03 nm (Figure 3 E), nicely fitting with the positive control and consistent with the previously reported binding constant of the wild‐type anti‐GFP‐enhancer nanobody.^13^, ^17^ Together, these results demonstrated that the photodeprotected photobody regains its full antigen binding affinity.

**Figure 3:**
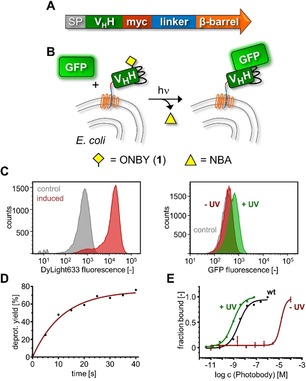
Determination of nanobody binding affinities using *E. coli* cell‐surface display and flow cytometry. A) Genetic fusion of anti‐GFP enhancer nanobody variants with outer membrane protein of the AIDA autodisplay system. SP=signal peptide. B) Scheme of *E. coli* presenting and binding nanobodies. NBA=nitrosobenzaldehyde. C) Flow cytometry analysis of *E. coli* cells presenting the ONBY–photobody after incubation with DyLight633‐coupled anti‐myc antibody (left panel) and after incubation with 10 nm sfGFP (right panel). Controls show uninduced cells that have not expressed a nanobody. D) Time‐course of photodeprotection of ONBY–photobody displayed on cells upon irradiation (*λ*=365 nm). E) Determination of binding constants.

We next aimed to demonstrate the potential of our novel anti‐GFP photobody for light‐controlled protein dimerization in a cellular assay. We envisioned a bispecific nanobody in which only one binding site is photocaged. We devised such nanobody–photobody fusion (construct **6**) consisting of the EgA1 nanobody, which is directed against domain 3 of the epidermal growth factor receptor (EGFR), and the anti‐GFP photobody, and obtained it by expression in *E. coli* (Figure 4). We transiently transfected HeLa cells with the transmembrane and extracellular domains of EGFR fused to the red‐fluorescent protein mCherry. We added the nanobody–photobody fusion **6** (10 nm) to the HeLa cells, allowed for binding of the anti‐EGFR nanobody portion of **6**, and then washed the cells. The cells were irradiated (20 s, *λ*=365 nm) to activate the photobody portion of **6**, whereas in control samples no irradiation was performed. We then added sfGFP (10 nm) and again washed the cells. Visualization of the cells by confocal fluorescent microscopy showed that binding of sfGFP could only be detected on transfected cells and with the photoactivated nanobody–photobody (Figure 4 D; see Figure S5 in the Supporting Information for the control experiment with the non‐caged bivalent nanobody construct **7**). Together, these results demonstrate that a photobody can be used in a cellular context and to design light‐dependent protein dimerizers based on a bispecific antibody.

**Figure 4:**
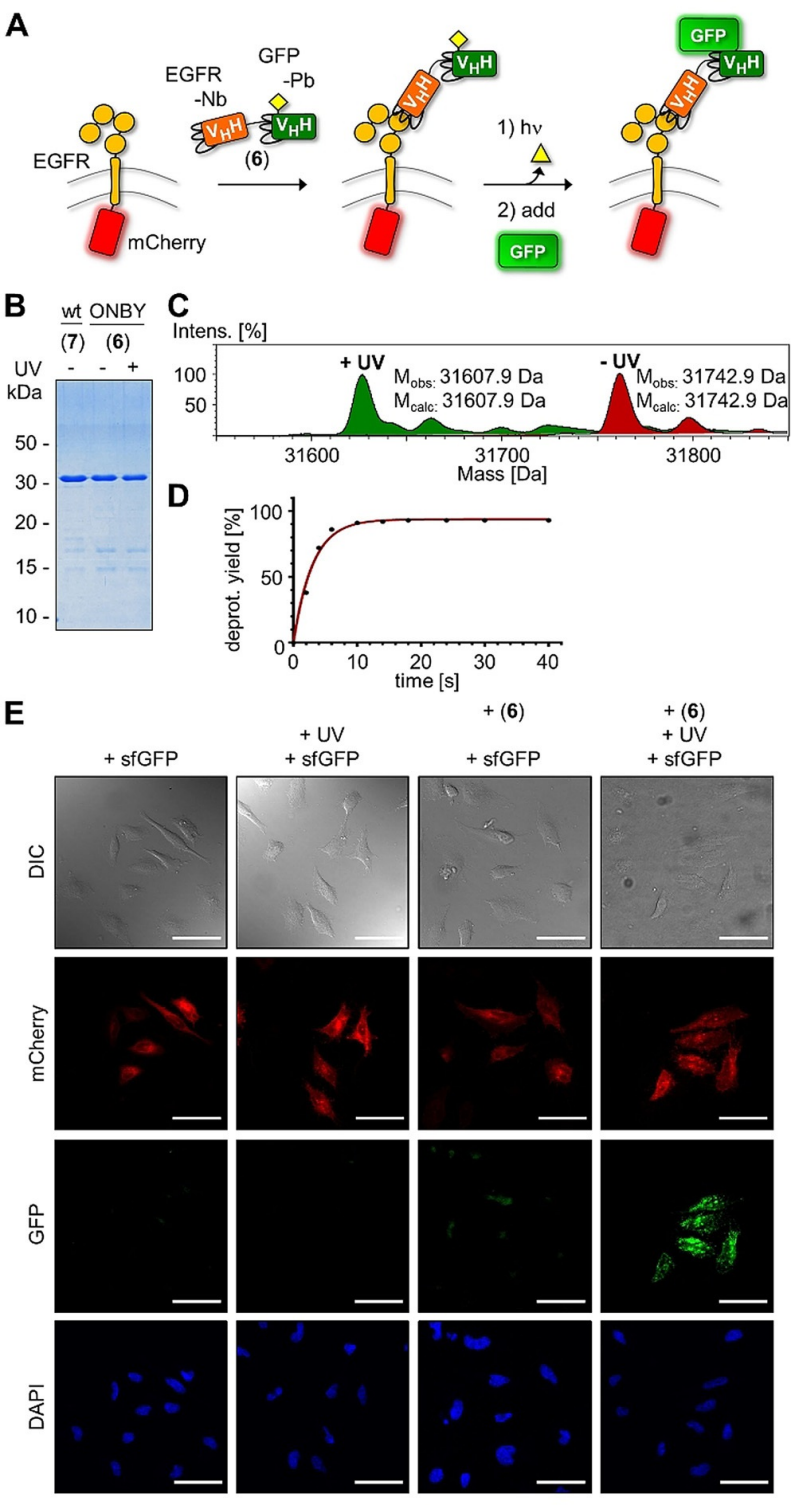
Extracellular binding assay with a bispecific nanobody–photobody (Nb–Pb) construct. A) Scheme of the assay. B) Coomassie‐stained SDS‐PAGE gel of bispecific fusion protein EgA1–enhancer(ONBY) (**6**). WT=wild‐type (**7**) protein containing Tyr instead of ONBY. C) ESI‐MS analysis of the photodeprotection reaction of **6** before (red) and after (green) irradiation with *λ*=365 nm. D) Time‐course of photodeprotection of **6** determined by ESI‐MS. E) Confocal microscopy images of HeLa cells transiently transfected with EGFR–mCherry and treated as illustrated in (A). Scale bar=50 μm.

Finally, we sought to generate more examples of our photobody design concept, including photobodies with potential therapeutic relevance. The aforementioned EgA1 nanobody binds to EGFR, which is upregulated or mutated in certain tumors. Two tyrosines in the nanobody, Tyr32 (in the CDR1 loop) and Tyr119 (at the end of the CDR3 loop), appeared highly promising for photocaging based on structural considerations (PDB: 4KRO; Supporting Information, Figure S6).^20^ We prepared a bispecific anti‐EGFR–anti‐GFP photobody–nanobody fusion (**8**), similar to construct **6**, however, this time with ONBY in the anti‐EGFR nanobody at position Tyr119 (Supporting Information, Figure S6). Indeed, the photocaged photobody–nanobody **8** did not bind to HeLa cells transfected with an EGFR–mCherry construct, but after photodeprotection, efficient binding to the transmembrane receptor could be monitored using confocal fluorescent microscopy (Supporting Information, Figure S7). We next selected the 2Rs15d nanobody, which binds domain 1 of human epidermal growth factor receptor 2 (HER2). HER2 is overexpressed in several types of breast cancer.^21^ We tested Tyr37 for our approach based on the crystal structure with the antigen (PDB: 5MY6;^21^ Supporting Information, Figure S8). A Y37ONBY–photobody–sfGFP fusion (**9**) was produced and labeled with Cy5. Prior to photodeprotection, the photobody (**9**) was unable to bind at detectable levels to BT‐474 cells overproducing the HER2 receptor; however, it specifically bound to these cells following light‐activation (Supporting Information, Figure S9). Finally, we chose another anti‐GFP nanobody (GFP‐minimizer; PDB: 3G9A).^13^ We identified Tyr113 in the CDR3 loop, which contacts the antigen only in a side‐on orientation, in contrast to the mostly pointed orientations found in the other examples presented (Supporting Information, Figure S10). The Y113ONBY‐photobody (**10**) exhibited a *K*_d_=2.28±0.03 μm for sfGFP, which is approximately 1000‐fold higher than the affinity reported for the wild‐type nanobody (Supporting Information, Figure S11 D).^13^ Photodeprotection reconstituted the binding activity of the photobody for the purified sfGFP antigen (Supporting Information, Figure S11) as well as binding of a Cy5‐labeled photobody **10** to GFP expressed as a fusion protein on the surface of HeLa cells (Supporting Information, Figure S12). Together, these data demonstrate the broad applicability of our photobody design concept and the versatile utility of the new protein reagents.

In conclusion, we report photoactivatable antibody molecules, termed photobodies, that were rationally designed with a single photocaging group in the binding region. The generation of four such photobodies and the frequent occurrence of suitable tyrosines in key positions of nanobody paratopes suggests this approach to be quite general. We expect this design concept to be extendable to other antibody and antibody‐like formats, such as full‐length IgG, scFv, diabodies, or monobodies, which similarly contain tyrosine as a frequent residue in their paratope regions,^22^ using either tyrosine or other side chains as caged entities. Photoactivatable antibody‐like molecules will find many applications derived from the binding, targeting, dimerization, and oligomerization properties of their uncaged parent proteins. Caging groups that are sensitive to longer wavelengths will be desirable in the future to enable reduced phototoxicity and deeper penetration of biological material. We have already shown the incorporation of NPY (**2**) with a red‐shifted absorption maximum compared to ONBY (**1**). Ultimately, even further red‐shifted or two‐photon decaging groups4b, 23 would open new avenues for applications in cell biology and live organisms, or even in patients. For example, we envision the combination of photobodies with concepts from antibody–drug conjugates or CAR‐T cells10c, 11a to achieve spatial and temporal control for these therapeutic strategies.

While this paper was under review, Sachdeva and co‐workers reported a similar design concept for the generation of an anti‐EGFR single‐domain antibody fragment (clone 7D12).^24^ Yu et al. developed optogenetically activatable split fragments of single‐domain antibody fragments (optobodies) for intracellular applications.^25^

## Conflict of interest

The authors declare no conflict of interest.

## Supporting information

As a service to our authors and readers, this journal provides supporting information supplied by the authors. Such materials are peer reviewed and may be re‐organized for online delivery, but are not copy‐edited or typeset. Technical support issues arising from supporting information (other than missing files) should be addressed to the authors.

Supplementary
