# Supplementary material for: Photobodies: Light‐Activatable Single‐Domain Antibody Fragments
Source: Angew Chem Int Ed Engl. 2019 Dec 12;59(4):1506–10. doi: 10.1002/anie.201912286 (PMC7004160; doi:10.1002/anie.201912286)
Supplement: Supplementary file 1 — Supplementary [file ANIE-59-1506-s001.pdf]

Angewandte Chemie
Eine Zeitschrift der Gesellschaft Deutscher Chemiker

Supporting Information

Photobodies: Light-Activatable Single-Domain Antibody Fragments

*Benedikt Jedlitzke, Zahide Yilmaz, Wolfgang Dörner, and Henning D. Mootz**

anie_201912286_sm_miscellaneous_information.pdf

SUPPORTING FIGURES

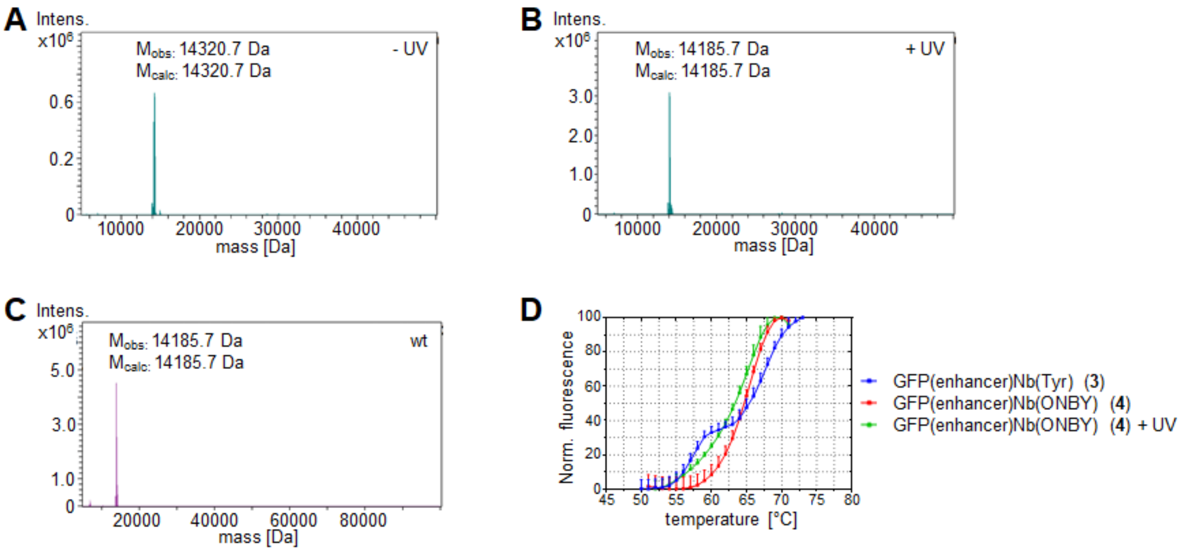

Figure S1. Further characterization of anti-GFP photobody(Y37ONBY) (**4**). A) & B) ESI-MS-analysis of purified protein before (A) and after (B) photo-deprotection. C) ESI-MS-analysis of corresponding wildtype anti-GFP enhancer nanobody (**3**). D) Thermal shift assay melting curves of wildtype (blue), photo-protected (red) and photo-deprotected (green) ONBY-nanobody.

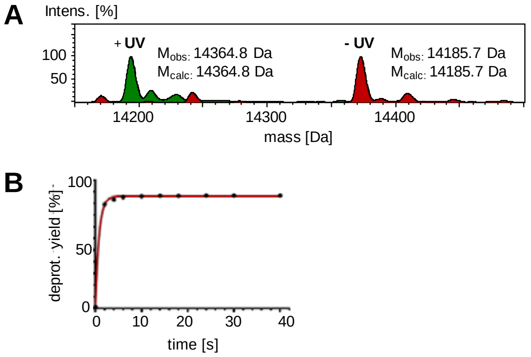

Figure S2. Further characterization of anti-GFP photobody(Y37NPY) (**5**). A) ESI-MS-analysis of purified anti-GFP photobody(Y37NPY) (**5**) before (red) and after (green) photo-deprotection. B) Time-course of photo-deprotection of the anti-GFP photobody(Y37NPY) (**5**) determined by ESI-MS analysis at the indicated time points.

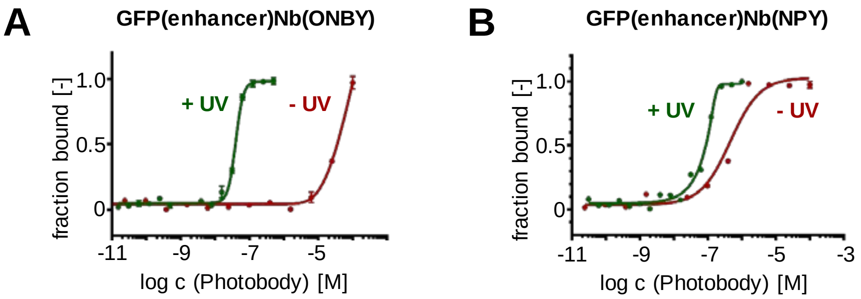

Figure S3. Microscale thermophoresis (MST) analysis of anti-GFP(enhancer) photobody (**4** and **5**) binding. This figure provides additional data to the experiments shown in Figure 2E. It additionally shows the analyses for the photo-deprotected photobodies. Note that the measurements for the decaged nanobodies were not quantitatively analyzable because the employed sfGFP concentration was more about 10 fold higher (10 nM) than the expected K_d to match the sensitivity of the MST device. This issue resulted in the asymmetric MST-curves. Nevertheless, this data shows the activation of the photobodies by photo-decaging.

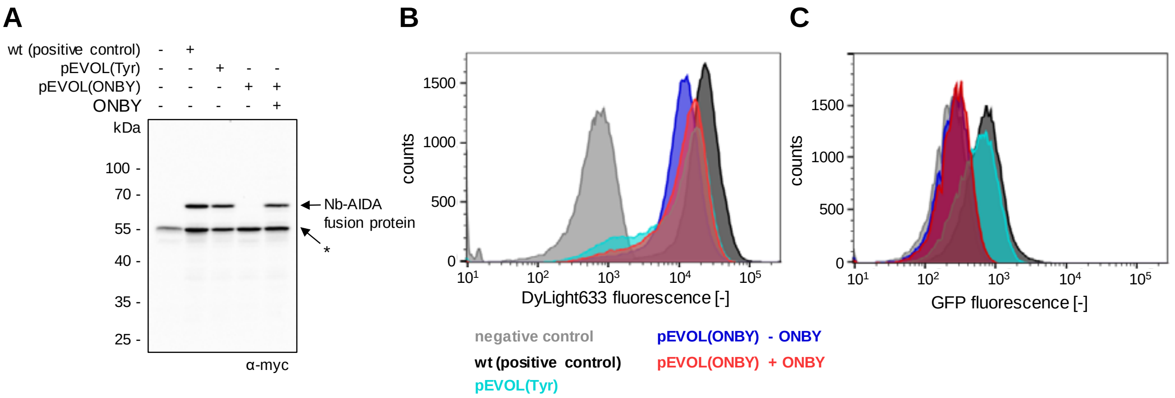

Figure S4. Bacterial cell surface display of anti-GFP photobody using the AIDA autodisplay system. *E. coli* cells carried different plasmids encoding nanobody-myc-tag-linker-AIDA fusion proteins: Plasmid GFP(enhancer)Nb-myc-AIDA for the positive and negative controls, or plasmid GFP(enhancer)Nb(Y37*TAG*)-myc-AIDA in combination with either a Tyr-specific *Mj*TyrRS (pEVOL(Tyr)) or an ONBY-specific *Mj*TyrRS (with or without addition of ONBY, as indicated). The negative control sample represents uninduced cells with the GFP(enhancer)Nb-myc-AIDA plasmid. A) Anti-myc western blot analysis of *E. coli* whole cell lysates prepared from *E. coli* cells that were carrying the indicated plasmids and were induced for protein expression with arabinose. The calculated molecular weights are: M(GFP(enhancer)Nb-AIDA) = 63.2 kDa; M(GFP(enhancer)Nb(ONBY)-AIDA) = 63.4 kDa. The asterisk indicates an unknown truncated protein. B) Flow cytometry analysis of arabinose induced *E. coli cells* following binding with a primary mouse anti-myc antibody and secondary DyLight633-labeled anti-mouse antibody. The presence of the myc tag on the cell surface is indicative of the presentation of the nanobody-AIDA fusion protein (see Figure 3A for sequence information). C) Flow cytometry analysis of arabinose induced *E. coli cells* following binding of sfGFP (10 nM).

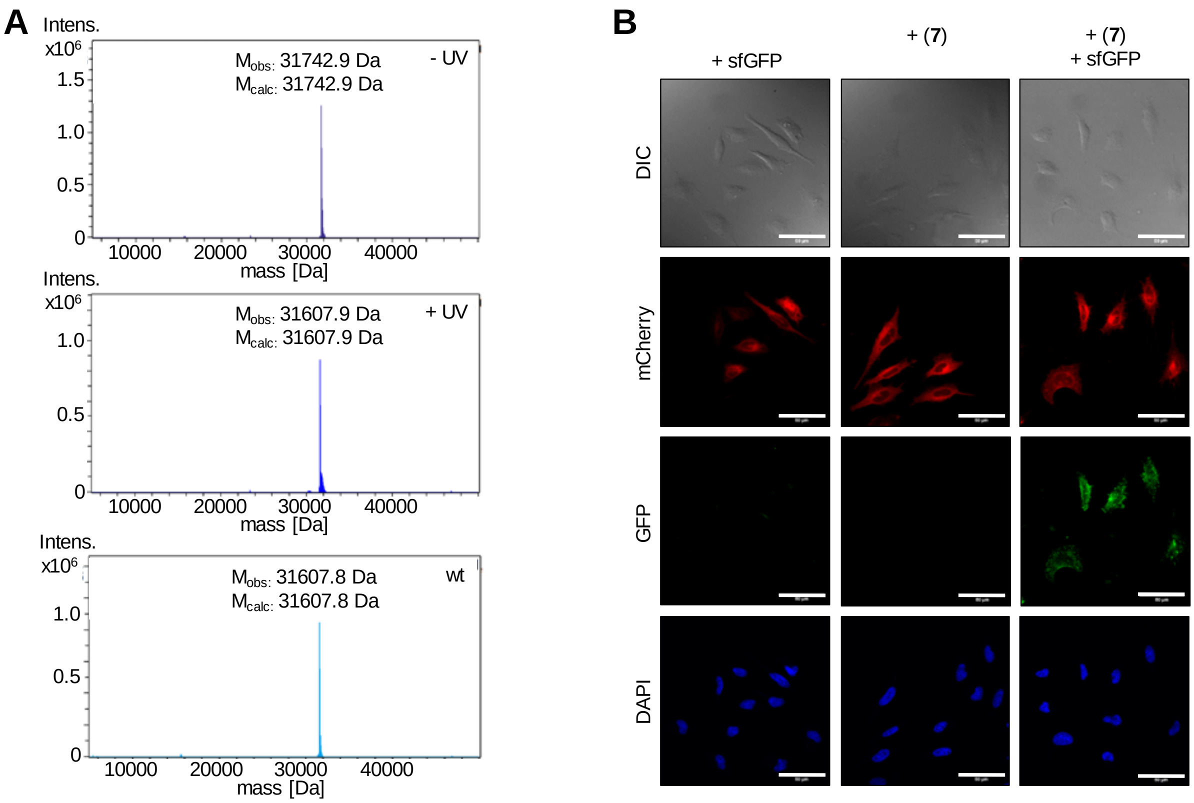

Figure S5. Characterization of bivalent antiEGFR-antiGFP(enhancer) nanobodies. This figure provides additional data to Figure 4. A) ESI-MS-analysis of the purified proteins antiEGFR-antiGFPenhancer(ONBY)-nanobody (**6**; top panels; with or without UV photo-deprotection) and antiEGFR-antiGFP(enhancer) (**7**) (bottom panel). B) Confocal microscopy images of HeLa cells transiently transfected with EGFR-mCherry, subsequently incubated with antiEGFR-antiGFP(enhancer) (**7**; 10 nM) and then sfGFP (10 nM), as indicated. White scale bar = 50 µM.

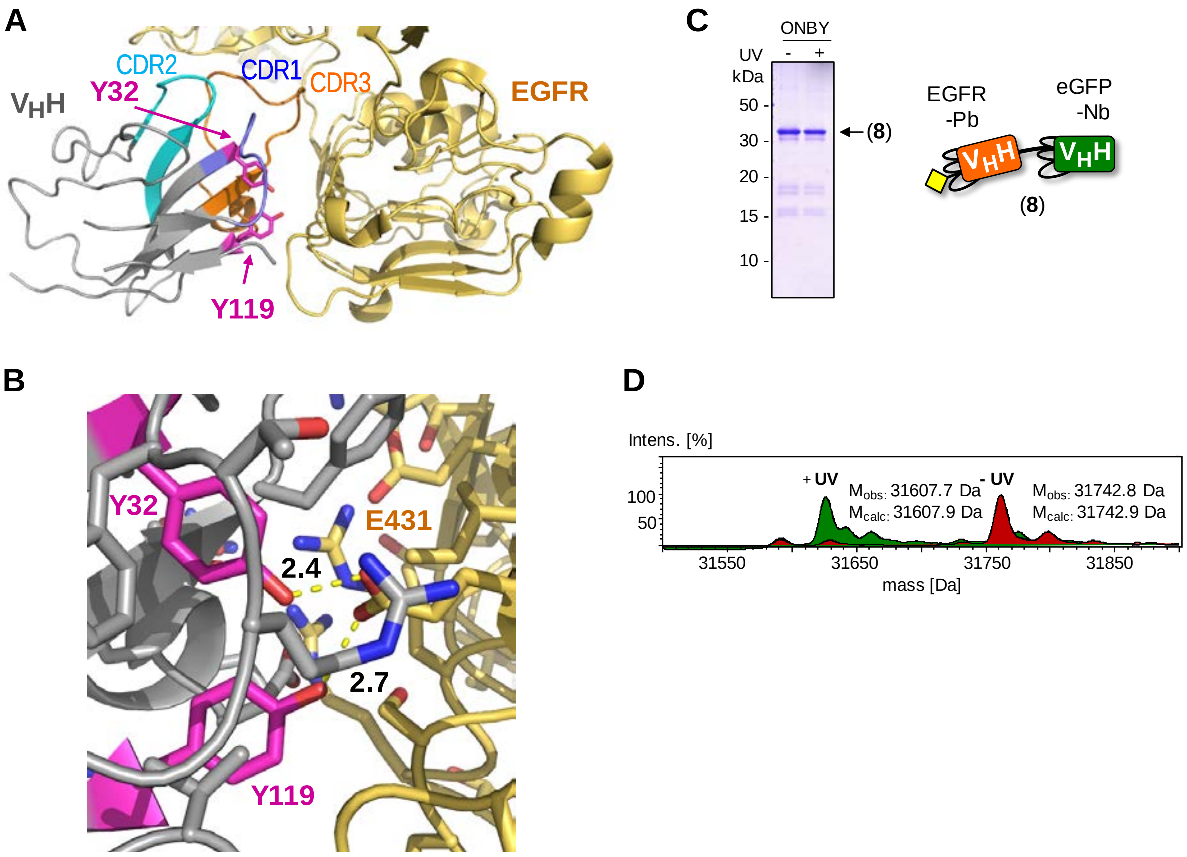

Figure S6. A photobody based on the EgA1 anti-EGFR nanobody. A) Complex between the EgA1 anti-EGFR nanobody and its antigen as seen in the crystal structure (pdb: 4KRO).[1] Tyr32 and Tyr119 are highlighted. B) Close-up of the structure shown in A) shows the embedding of Tyr32 and Tyr119 at the interface. Black numbers indicate distances in Ångström for the indicated residues. C) Coomassie-stained SDS-PAGE gel of the purified dimeric nanobody antiEGFR(Y119ONBY)-antiGFP(enhancer) (**8**) before and after UV irradiation. M(calc.) = 31.7 kDa. D) ESI-MS-analysis of the purified dimeric nanobody antiEGFR(Y119ONBY)-antiGFP(enhancer) (**8**) before and after UV irradiation.

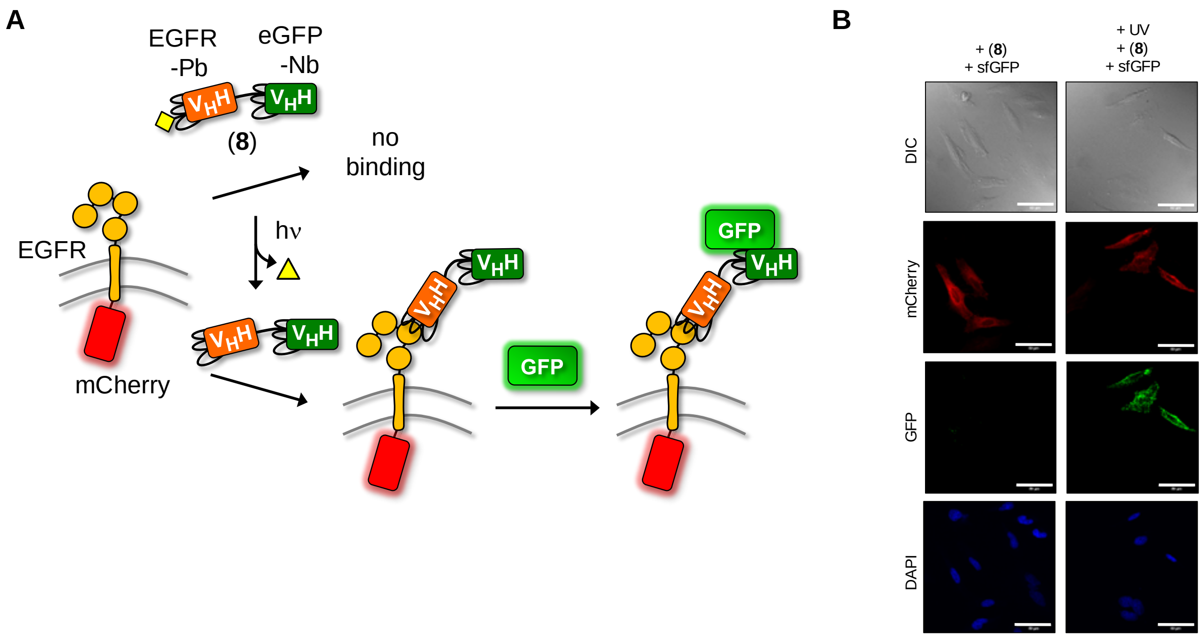

Figure S7. Cellular binding assay using bivalent antiEGFR(Y119ONBY)-antiGFP(enhancer) (**8**). A) Scheme of the assay. B) Confocal microscopy images of HeLa cells transiently transfected with EGFR-mCherry, incubated with antiEGFR(Y119ONBY)-antiGFP(enhancer) (**8**; 10 nM), which was or was not UV-irradiated, and then incubated with sfGFP (10 nM), as indicated. White scale bar = 50 µM.

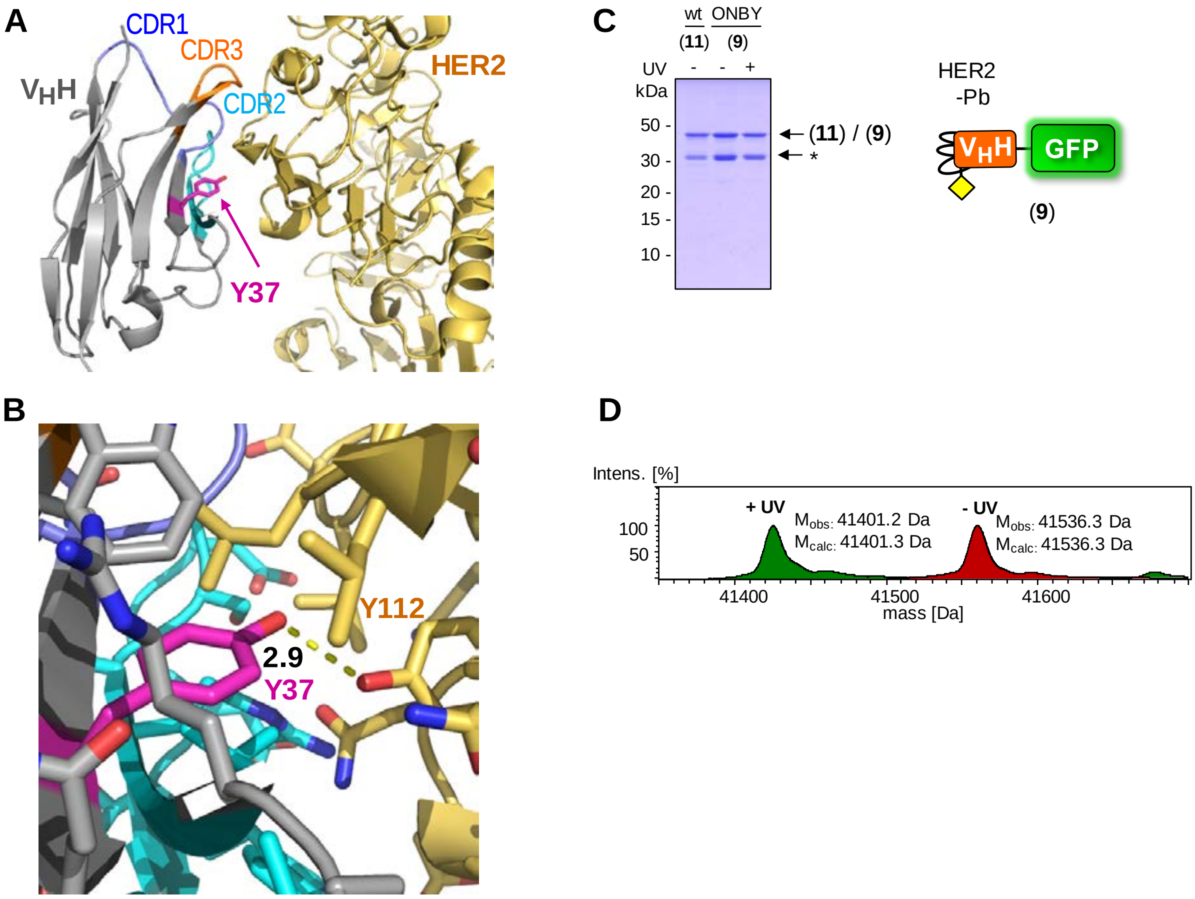

Figure S8. A photobody based on the 2Rs15d anti-HER2 nanobody. A) Complex between the 2Rs15d anti-HER2 nanobody and its antigen as seen in the crystal structure (pdb: 5MY6).[2] Tyr37 is highlighted. B) Close-up of the structure shown in A) shows the embedding of Tyr37 at the interface. The black number indicates the distance in Ångström for the indicated residues. C) Coomassie-stained SDS-PAGE gel of the purified nanobody-sfGFP fusion antiHER2(Y37ONBY)-sfGFP (**9**) before and after UV irradiation. M(calc.) = 41.5 kDa. Shown for comparison is the wildtype antiHER2-sfGFP fusion protein (**11**). The band marked with an asterisk is a truncated protein contamination consisting of sfGFP as the band was green fluorescent without heat-denaturation of the sample (data not shown). D) ESI-MS-analysis of antiHER2(Y37ONBY)-sfGFP (**9**) before and after UV irradiation.

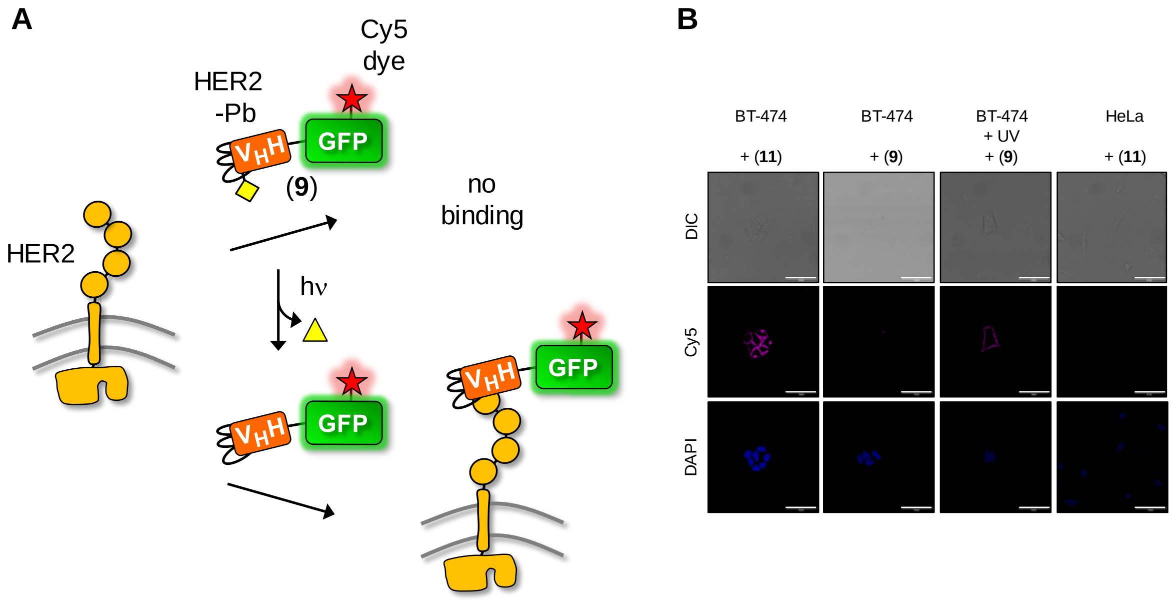

Figure S9. Cellular binding assay using antiHER2(Y37ONBY)-sfGFP (**9**). The photobody antiHER2(Y37ONBY)-sfGFP (**9**) was randomly labeled with Cy5 on lysine side chains using Cy5-NHS. A) Scheme of the assay. B) Confocal microscopy images of BT-474 cells overexpressing the HER2 receptor. The Cy5-labeled photobody antiHER2(Y37ONBY)-sfGFP (**9**; 10 nM) was either used in caged form or photo-decaged by UV-irradiation prior to its addition to cells. Untransfected HeLa cells were used as a negative control for binding (panels on the right). White scale bar = 50 µM.

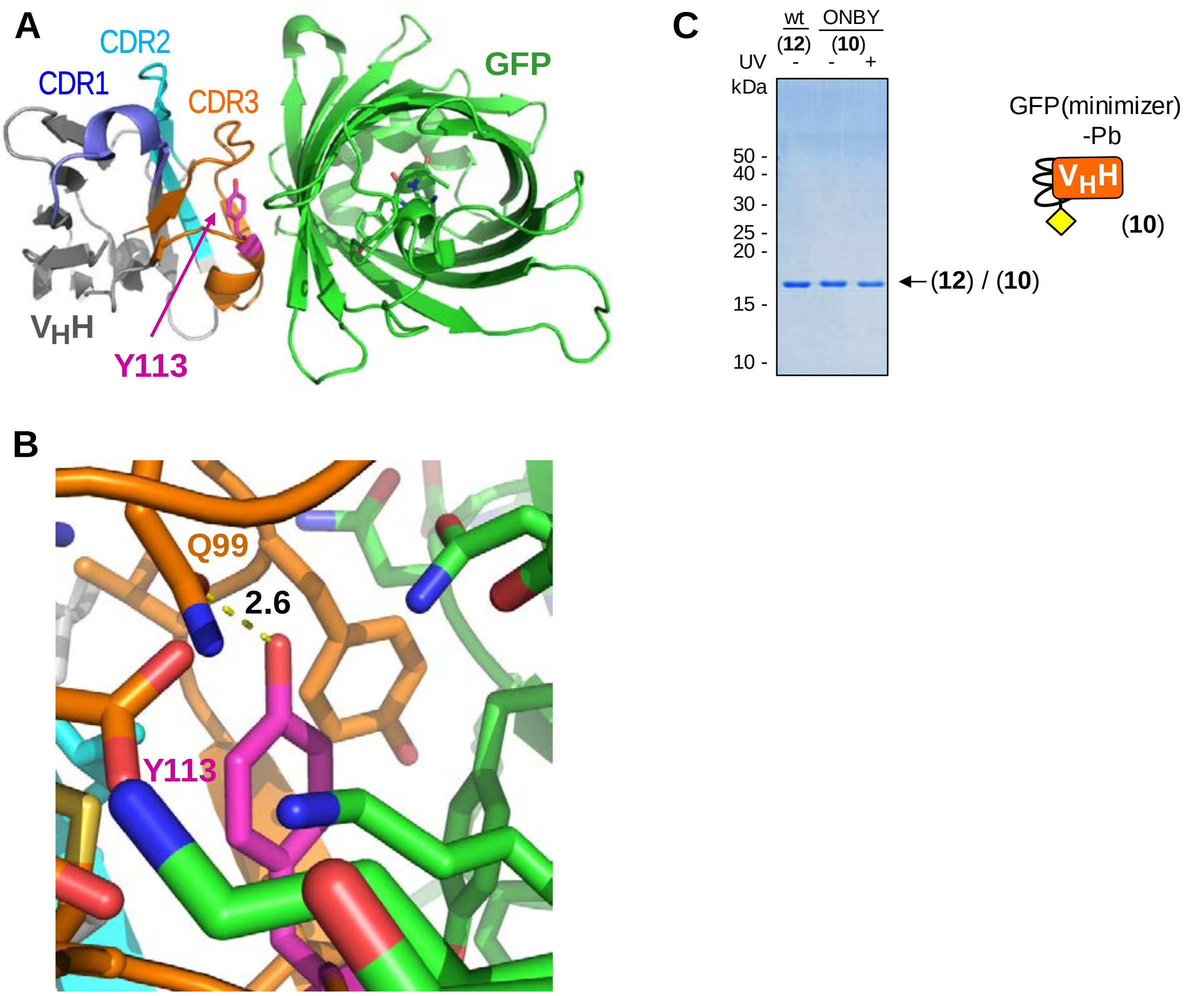

Figure S10. A photobody based on the minimizer antiGFP nanobody. A) Complex between the minimizer anti-GFP nanobody and its antigen as seen in the crystal structure (pdb: 3G9A).[3] Tyr113 is highlighted. B) Close-up of the structure shown in A) shows the embedding of Tyr113 at the interface. The black number indicates the distance in Ångström for the indicated residues. C) Coomassie-stained SDS-PAGE gel of the purified photobody antiGFP(Y113ONBY) (**10**) before and after UV irradiation. M(calc.) = 16.5 kDa. Shown for comparison is the wildtype antiGFPminimizer protein (**12**).

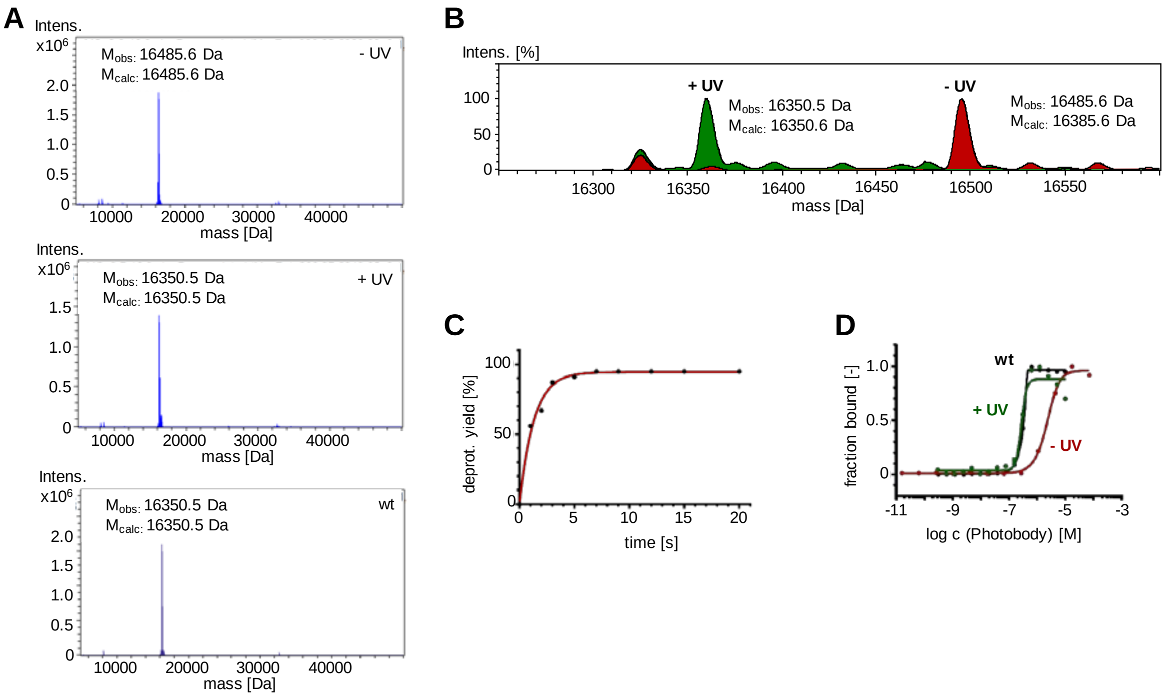

Figure S11. Characterization of the photobody antiGFPminimizer(Y113ONBY) (**10**). A) ESI-MS-analysis of the purified photobody antiGFPminimizer(Y113ONBY) (**10**; top panels; with or without UV photo-deprotection) and the parent antiGFPminimizer nanobody (**12**) (bottom panel). B) ESI-MS-analysis of antiGFPminimizer(Y113ONBY) (**10**) before (red) and after (green) photo-decaging by UV-irradiation (λ = 365 nm). C) Time-course of photo-deprotection. D) Determination of binding affinity of antiGFPminimizer(Y113ONBY) (**10**) before (red) and after (green) photo-deprotection using microscale thermophoresis (MST). sfGFP (10 nM) was mixed with a dilution series of the respective nanobodies. Note that these measurements were not quantitatively analyzable for the decaged nanobody and the wildtype control because the employed sfGFP (antigen) concentration was about 10 fold higher than the expected K_d to match the sensitivity of the MST device. This technical problem also resulted in the asymmetric MST-curves. Nevertheless, this data shows the activation of the photobody **10** by photo-decaging.

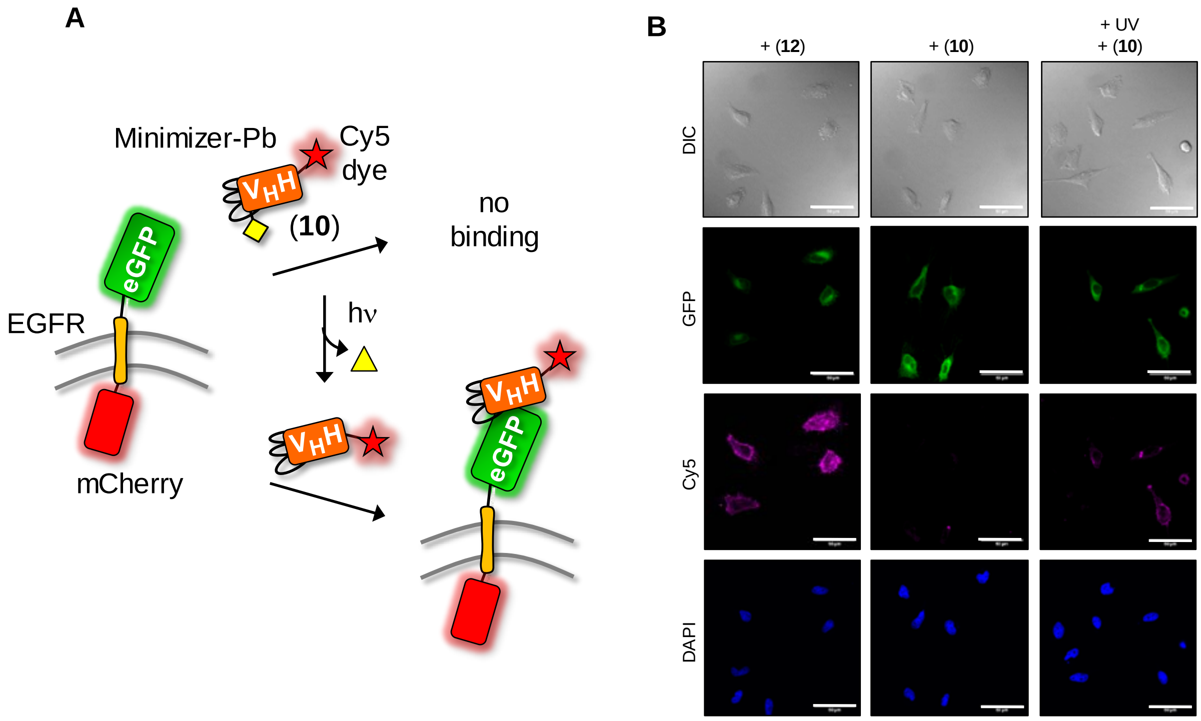

Figure S12. Cellular binding assay using the antiGFPminimizer(Y113ONBY) photobody (**10**). The photobody antiGFPminimizer(Y113ONBY) photobody (**10**) was randomly labeled with Cy5 on lysine side chains using Cy5-NHS. A) Scheme of the assay. B) Confocal microscopy images of HeLa cells transiently transfected to express HA-eGFP-Trx-TMD-mCherry (TMD = transmembrane domain of the PDGF receptor). The Cy5-labeled photobody antiGFPminimizer(Y113ONBY) photobody (**10**; 10 nM) was either used in caged form or photo-decaged by UV-irradiation prior to its addition to cells. The left panels show a control experiment using the wildtype antiGFPminimizer nanobody. White scale bar = 50 µM.

MATERIALS AND METHODS

General

Antibiotics were used at the following concentrations: 100 µg/mL ampicillin, 34 µg/mL chloramphenicol, 50 µg/mL kanamycin. For UV-irradiation an LED lamp M365LP1-365 nm with 1400 mA was used (Thorlabs Inc, Newton, USA). Samples were irradiated for the indicated time periods with an irradiation distance of 10 cm.

Recombinant gene expression and protein purification:

Table S1 shows the list of nanobody proteins produced in this study and their encoding expression plasmids. *E. coli* BL21(DE3) Gold cells were transformed with the respective expression plasmid without amber stop codons for suppression in their encoding genes. For amber stop codon suppression *E. coli* K12(DE3) UT5600 cells[4] were used and were co-transformed with the respective expression plasmid and the pEVOL-ONBY plasmid. Cells were cultured at 37 °C in LB-medium (600 mL) with the corresponding antibiotic(s) (100 µg/mL ampicillin, 34 µg/mL chloramphenicol) until an OD600 of 0.7 – 0.9 was reached. The temperature was then shifted to 28 °C and protein expression was induced for 4 h by adding IPTG (0.4 mM final concentration). For suppression conditions, 1 mM o-(2-nitrobenzyl)-L-tyrosine (ONBY) or nitropiperonyl-L-tyrosine (NPY), presolved in 1 M NaOH, and 0.2% arabinose were added, and cells were incubated for 4 h at 37 °C. Cells were pelleted by centrifugation and resuspended in Ni-NTA buffer (50 mM Tris/HCl, 300 mM NaCl, pH 8.0). Resuspended cells were ruptured using an Emulsiflex C5 emulsifier (Avestin). Insoluble material was removed by centrifugation and the supernatant fractions were used to purify the proteins. Purification of His-tagged proteins *via* Ni-NTA affinity chromatography was performed at 4 °C using flow gravity flow columns with a bed volume of 1.0 mL of Ni-NTA resin (Cube Biotech) pre-equilibrated with Ni-NTA buffer with 20 mM imidazole. Following loading of the supernatant fractions (with 20 mM imidazole), three steps of washing with five to ten column volumes (cv) of Ni-NTA buffer (with 20 mM imidazole) were performed. Proteins were eluted with 4 mL Ni-NTA buffer (with 250 mM imidazole) fractions containing the desired protein were pooled and concentrated in Vivaspin® Turbo 4 concentrator spin columns (Sartorius, Göttingen, Germany) to ~¼ of the volume.

As a second purification step a size exclusion chromatography was performed on an ÄKTA Purifier System (GE Healthcare) with a Superdex200 column and a flow rate of 1 mL/min in PBS buffer (140 mM NaCl, 2.7 mM KCl, 1.5 mM KH_2PO_4, 8.1 mM Na_2HPO_4, pH 7.4). Protein elution was monitored by absorption at 280 nm and collected fractions were analyzed by SDS-PAGE. Purified fractions were combined, 10% glycerol (v/v) was added, concentrated in Vivaspin® Turbo 4 concentrator spin columns (Sartorius, Göttingen, Germany), flash frozen in liquid nitrogen and stored at -80 °C. Protein concentrations were determined using the calculated extinction coefficient at 280 nm.

Cy5 labeling of nanobody constructs

A 10 µM solution of respective nanobodies in PBS buffer (pH 8) was incubated with 30 µM final concentration of Cy5-NHS-ester (GEPA15101; GE Healthcare) for 2 h at room temperature. Afterwards, the unreacted dye was dialyzed out against PBS buffer (pH 7.4) and the concentration was determined using absorption at 649 nm and an extinction coefficient of 250,000 $cm^{-1}M^{-1}$.

Nanobody display on cell surface

Nanobody encoding genes were cloned into a pBAD-vector for the AIDA autodisplay system[5] using *E. coli* DH5α cells as cloning host. *E. coli* BL21 Gold (DE3) cells were co-transformed with the respective AIDA plasmid (Table S2) and, if applicable, with the pEVOL-ONBY plasmid for incorporation of o-(2-nitrobenzyl)-L-tyrosine (ONBY) or nitropiperonyl-L-tyrosine (NPY). Presenting *E. coli* cells were prepared by inoculating LB medium (20 mL) with respective antibiotics with an overnight culture in the ratio 1:20. Bacterial cell cultures were then grown at 37 °C with shaking (180 rpm) until an OD600 of 0.5 was reached. In case of suppression conditions, ONBY or NPY (each at 3 mM final concentration) was added to the medium and gene expression was induced with 0.4% arabinose. Induced cells were cultured for 3 h at 30 °C in case of regular expression without amber stop codon suppression or for 2 h at 37 °C in case of suppression with the unnatural amino acid. The cells were then washed three times with ice cold PBS buffer (140 mM NaCl, 2.7 mM KCl, 1.5 mM KH_2PO_4, 8.1 mM Na_2HPO_4, pH 7.4; sterile-filtered) using a centrifugation step (5000 *g*) at 4 °C for 2 min to pellet the cells, and then stored after resuspension in 1 mL PBS buffer at 4 °C overnight.

Determination of binding affinity of nanobodies presented on cell surface by flow cytometry

Nanobody presenting *E. coli* cells were washed with ice cold PBS buffer (140 mM NaCl, 2.7 mM KCl, 1.5 mM KH_2PO_4, 8.1 mM Na_2HPO_4, pH 7.4; sterile-filtered), pelleted by centrifugation (5000 *g* for 2 min at 4 °C) and were then resuspended in PBS buffer and diluted to OD600 = 1.0. For photo-deprotection, 100 µL cells (OD600 = 1) were transferred into a thin-walled PCR tube (Greiner Bio-One, Kremsmuenster, Austria) and irradiated for 45 sec. For binding of sfGFP, a total volume of 100 µL cell suspension (OD600 = 1) were pelleted, resuspended in sfGFP solutions of different concentrations (each 40 µL) and then incubated for 20 min at 25 °C. For detection of the myc-tag, cells were resuspended in 50 µL PBS, 0.5 µL primary monochlonal anti-myc antibody (MA1-21316, Thermo Fisher) was added and incubated for 30 min at 25 °C. After washing two times with 200 µL PBS, cells were resuspended in 50 µL PBS and 0.5 µL secondary antibody anti-mouse-DyLight633 antibody (10006103; Thermo Fisher) was added and incubated for 20 min at 25 °C. Subsequently, 200 µL PBS was added and the cells were washed three times with 200 µL PBS each. Cells were stored in dark on ice until flow cytometry analysis was carried out.

Prior to analysis by flow cytometry, the samples were diluted ten-fold with PBS buffer. Flow cytometry measurements were performed with a FACS Aria III (BD, Heidelberg, Germany) using a 488 nm laser for excitation and 530/30 BP- and 502 LP-filter for detection of GFP (698 V). For DyLight633 detection, a 633 nm laser was used for excitation in addition to a 660/20 BP filter detection (852 V). For each sample, a total of 50,000 cells were recorded at a rate of 2,000 cells per second and analyzed by FACS DIVA 8.0 software (BD, Heidelberg, Germany). The forward and sideward scattering was detected with 300 V. Measured mean fluorescence of samples were plotted against the log (concentration) of sfGFP and K_D determined by nonlinear fitting with Origin2019 software (OriginLab Corporation, Northampton, US).

Microscale thermophoresis (MST)

A dilution series with 16 aliquots of the nanobody was prepared in PBS buffer before measurement. 10 µL of fluorescent sfGFP protein (20 nM) was mixed with 10 µL of the 16 nanobody aliquots and incubated for 15 min at 25 °C. MST measurements were performed on a Nanotemper Monolith NT.115 with standard coated capillaries (NanoTemper Technologies, Munich, Germany). The blue LED power was at 100% and the MST power was at 80%. Measured data points were exported and plotted against the log (concentration) of nanobody to determine K_D by nonlinear fitting with Origin2019 software (OriginLab Corporation, Northampton, US). For measurements of UV-deprotected samples, 100 µL of photobody was transferred into a thin-walled PCR tube (Greiner Bio-One, Kremsmuenster, Austria) and irradiated (365 nm) for 15 sec.

Thermal shift assay

For measurements of UV-deprotected samples, the photobody was transferred into a thin-walled PCR tube (Greiner Bio-One, Kremsmuenster, Austria) and irradiated (365 nm) for 15 sec. After deprotection the protein solution was dialyzed against PBS.

Protein solutions and SYPRO™ Orange (stock solution of 5000x) were added to the wells of a 96-well PCR plate with final concentrations of 5 mg/ml and 5x. The sealed PCR plate was placed into the CFX96 Touch™ Real-Time PCR Detection System (Bio-Rad, Hercules, USA). In increments of 1 °C samples were heated from 10 to 90 °C while the fluorescence was measured after each cycle. Using GraphPad Prism 3 (GraphPad, San Diego, USA) the truncated fluorescence data was normalized and fitted to a Boltzmann sigmoidal curve. Measurements were performed 3 times for each protein.

$$y = \mathrm{bottom} + \frac{\mathrm{top} - \mathrm{bottom}}{1 + \exp\left(\frac{T_m - x}{\mathrm{slope}}\right)}$$

Cell culture

HeLa cells were cultured in EMEM (supplemented with 10% fetal calf serum, 1% non-essential amino acids and 1% L-glutamine) at 37 °C and 5% CO_2. Confluent cells were used for transient transfection

with the respective plasmid (see Table S3), encoding either HA-EGFR-mCherry or HA-eGFP-Trx-TMD-mCherry (TMD = transmembrane domain of the PDF receptor), using lipofectamine 2000 (Invitrogen) as recommended by the manufacturer. After removal of lipofectamine 2 x 10^5 cells were seeded on 24 mm coverslips in a 35 mm dish. Following 36 h of incubation, binding studies and microscopy analysis were performed. To this end, cells were washed two times with PBS and purified photobodies were added in fresh medium for 20 min at 37 °C. To cells transfected with HA-EGFR-mCherry either 10 nM bispecific nanobody-photobody (EgA1Nb-GFP(enhancer)Nb(Y37ONBY)) or 10 nM bispecific photobody-nanobody (EgA1Nb(Y119ONBY)-GFP(enhancer)Nb) was added. To cells transfected with HA-eGFP-Trx-TMD-mCherry 10 nM of the anti-GFP minimizer photobody was added. Subsequently, cells were washed again with PBS and then incubated with 10 nM sfGFP in fresh medium for 5 min at 37 °C. Finally, cells were washed three times with PBS.

For UV deprotection of the nanobody-photobody (EgA1Nb-GFP(enhancer)Nb(Y37ONBY)) protein bound to cells, the coverslips were irradiated (365 nm) for 20 sec prior to addition of sfGFP.

For UV deprotection of the bispecific photobody-nanobody (EgA1Nb(Y119ONBY)-GFP(enhancer)Nb) and the anti-GFP minimizer photobody the same procedure as described above for the anti-GFP enhancer photobody was applied prior to the addition of the decaged photobody constructs to cells.

BT-474 cells were cultured in RPMI 1640 (supplemented with 20% fetal calf serum, 10 µg/mL human insulin and 2 mM L-glutamine) at 37 °C and 5% CO_2. 1 x 10^5 cells were seeded on 24 mm coverslips in a 35 mm dish and cultured until high confluency was reached. For binding studies and microscopy analysis the cells were washed two times with PBS and 40 nm of purified nanobody (HER2Nb-sfGFP-H_6-Cy5) or photobody (HER2Nb(Y37ONBY)-sfGFP-H_6-Cy5) was added in fresh medium for 15 min at 37 °C. Afterwards cells were washed three times with PBS.

For UV deprotection, prior the addition to cells, the photobody (HER2Nb(Y37ONBY)-sfGFP-H_6-Cy5) was irradiated for 15 sec in a thin-walled PCR tube (Greiner Bio-One, Kremsmuenster, Austria).

Confocal laser scanning microscopy

Cells were fixed with 4% paraformaldehyde in PBS for 20 min at 25 °C, washed three times with PBS and mounted on coverslips using Aqua/Poly-Mount mounting solution (Polysciences). Confocal microscopy was carried out using a 63X water-immersion objective lens on a Leica DMi8 system.

Mass spectrometry

Mass analyses of intact proteins were performed using an UltiMate™ 3000 RS system (Thermo Fisher Scientific GmbH, Dreieich, Germany) connected to a maXis II UHR-qTOF mass spectrometer (Bruker Daltonik GmbH, Bremen, Germany) with a standard ESI source (Apollo, Bruker Daltonik GmbH, Bremen, Germany). Proteins were reduced with 2 mM TCEP at rt for 10 minutes to avoid inhomogeneity

issues. Then, samples were acidified using a 5% formic acid solution to reach a pH 2-3 and centrifuged (14000 rpm, 3 min). According to the protein concentration, an appropriate volume of the supernatant was loaded on a C4 column (Advance Bio RP-mAb C4, 2.1 mm x 50 mm, 3.5 µm, Agilent Technologies, Waldbronn, Germany) at a flow rate of 0.6 mL/min in 5% eluent B (eluent A: 0.1% formic acid in water; eluent B: 0.1% formic acid in acetonitrile). After a desalting period of 7 minutes at 5% B, a steep gradient was applied (5-60% B in 2 min). MS settings: capillary voltage 4500 V, end-plate offset 500 V, nebulizer 5.0 bar, dry gas 9.0 L/min, dry T=200°C, mass range m/z 300-3000. Data were analyzed with DataAnalysis 4.4 (Bruker Daltonik GmbH, Bremen, Germany) and deconvolution was performed using the MaxEnt algorithm implemented in the software. Unless otherwise mentioned, the averagine-based SNAP algorithm was employed to identify peaks and to calculate the monoisotopic masses.

SUPPORTING TABLES

Table S1. List of purified recombinant nanobody constructs and their expression plasmids

Protein number	Name of construct	Encoding plasmid	Vector backbone
**3**	GFP(enhancer)Nb(Y37Tyr)*-H_6	pBJ167 + pEVOL(Tyr)	pET22b(+)
**4**	GFP(enhancer)Nb(Y37ONBY)-H_6	pBJ167 + pEVOL(ONBY)	pET22b(+)
**5**	GFP(enhancer)Nb(Y37NPY)-H_6	pBJ167 + pEVOL(ONBY)	pET22b(+)
**6**	EgA1Nb-GFP(enhancer)Nb(Y37ONBY)-H_6	pBJ200 + pEVOL(ONBY)	pET22b(+)
**7**	EgA1Nb-GFP(enhancer)Nb-H_6	pBJ201	pET22b(+)
**8**	EgA1Nb(Y119ONBY)-GFP(enhancer)Nb-H_6	pZY94 + pEVOL(ONBY)	pET22b(+)
**9**	HER2Nb(Y37ONBY)-sfGFP-H_6	pZY70 + pEVOL(ONBY)	pET28a (+)
**10**	GFP(minimizer)Nb(Y116ONBY)-H_6	pBJ205 + pEVOL(ONBY)	pET22b(+)
**11**	HER2Nb-sfGFP-H_6	pZY56	pET28a (+)
**12**	GFP(minimizer)Nb-H_6	pBJ204	pET22b(+)

Table S2. List of plasmids used for nanobody presentation in the AIDA autodisplay system

Protein number (when mentioned in text and figures)	Name of construct	Encoding plasmid	Vector backbone
-	GFP(enhancer)Nb-myc-AIDA	pBJ72	pBAD[5]
-	GFP(enhancer)Nb(Y37ONBY)-myc-AIDA	pBJ129	pBAD[5]
-	GFP(enhancer)Nb(Y37NPY)-myc-AIDA	pBJ129	pBAD[5]

Table S3. List of plasmids used for cell culture experiments

Protein number (when mentioned in text and figures)	Name of construct	Encoding plasmid	Vector backbone
-	HA-EGFR-mCherry	pMBH65	pDisplay
-	HA-eGFP-Trx-TMD-mCherry	pMBH63	pDisplay

Amino acid sequences of purified recombinant nanobody constructs

(3) GFP(enhancer)Nb(Y37Tyr)-H6

MAQVQLVESGGALVQPGGSLRLSCAASGFPVNRYSMRWYRQAPGKEREWVAGMSSAGDRSSYEDSVKGRFTISR
DDARNTVYLQMNSLKPEDTAVYYCNVNVGFEYWGQGTQVTVSSPDRSHHHHHH

(4) GFP(enhancer)Nb(Y37ONBY)-H6

MAQVQLVESGGALVQPGGSLRLSCAASGFPVNRYSMRW**Y(37ONBY)**RQAPGKEREWVAGMSSAGDRSSYEDSVK
GRFTISRDDARNTVYLQMNSLKPEDTAVYYCNVNVGFEYWGQGTQVTVSSPDRSHHHHHH

(5) GFP(enhancer)Nb(Y37NPY)-H6

MAQVQLVESGGALVQPGGSLRLSCAASGFPVNRYSMRW**Y(37NPY)**RQAPGKEREWVAGMSSAGDRSSYEDSVKG
RFTISRDDARNTVYLQMNSLKPEDTAVYYCNVNVGFEYWGQGTQVTVSSPDRSHHHHHH

(6) EgA1Nb-GFP(enhancer)Nb(Y37ONBY)-H6

MGQVQLQESGGGLVQPGGSLRLSCAASGRTFSSYAMGWFRQAPGKQREFVAAIRWSGGYTYYTDSVKGRFTISRD
NAKTTVYLQMNSLKPEDTAVYYCAATYLSSDYSRYALPQRPLDYDYWGQGTQVTVSSLEWAQSGGGGSSSASGG
SGGSAQVQLVESGGALVQPGGSLRLSCAASGFPVNRYSMRW**Y(37ONBY)**RQAPGKEREWVAGMSSAGDRSSYED
SVKGRFTISRDDARNTVYLQMNSLKPEDTAVYYCNVNVGFEYWGQGTQVTVSSPDGSESGDYKDDDDKHHHHHH

(7) EgA1Nb-GFP(enhancer)Nb-H_6

MGQVQLQESGGGLVQPGGSLRLSCAASGRTFSSYAMGWFRQAPGKQREFVAAIRWSGGYTYYTDSVKGRFTISRD
NAKTTVYLQMNSLKPEDTAVYYCAATYLSSDYSRYALPQRPLDYD**Y**WGQGTQVTVSSLEWAQSGGGGSSSASGG
SGGSAQVQLVESGGALVQPGGSLRLSCAASGFPVNRYSMRW**Y**RQAPGKEREWVAGMSSAGDRSSYEDSVKGRFTI
SRDDARNTVYLQMNSLKPEDTAVYYCNVNVGFEYWGQGTQVTVSSPDGSESGDYKDDDDKHHHHHH

(8) EgA1Nb(Y119ONBY)-GFP(enhancer)Nb-H_6

MGQVQLQESGGGLVQPGGSLRLSCAASGRTFSSYAMGWFRQAPGKQREFVAAIRWSGGYTYYTDSVKGRFTISRD
NAKTTVYLQMNSLKPEDTAVYYCAATYLSSDYSRYALPQRPLDYD**Y(119ONBY)**WGQGTQVTVSSLEWAQSGGG
GSSSASGGSGGSAQVQLVESGGALVQPGGSLRLSCAASGFPVNRYSMRWYRQAPGKEREWVAGMSSAGDRSSYE
DSVKGRFTISRDDARNTVYLQMNSLKPEDTAVYYCNVNVGFEYWGQGTQVTVSSPDGSESGDYKDDDDKHHHHH
H

(9) HER2Nb(Y37ONBY)-sfGFP-H_6

MGQVQLQESGGGSVQAGGSLKLTCAASGYIFNSCGMGW**Y(37ONBY)**RQSPGRERELVSRISGDGDTWHKESVKGR
FTISQDNVKKTLYLQMNSLKPEDTAVYFCAVCYNLETYWGQGTQVTVSSGGGSGGGSASSKGEELFTGVVPILVEL
DGDVNGHKFSVRGEGEGDATNGKLTLKFICTTGKLPVPWPTLVTTLTYGVQCFSRYPDHMKRHDFFKSAMPEGYV
QERTISFKDDGTYKTRAEVKFEGDTLVNRIELKGIDFKEDGNILGHKLEYNFNSHNVYITADKQKNGIKANFKIRHN
VEDGSVQLADHYQQNTPIGDGPVLLPDNHYLSTQSVLSKDPNEKRDHMVLLEFVTAAGITHGGSESGDYKDDDDK
HHHHHH

(10) GFP(minimizer)Nb(Y116ONBY)-H_6

MGADVQLQESGGGSVQAGGSLRLSCAASGDTFSSYSMAWFRQAPGKECELVSNILRDGTTTYAGSVKGRFTISRDD
AKNTVYLQMVNLKSEDTARYYCAADSGTQLGYVGAVGLSCLD**Y(116ONBY)**VMDYWGKGTQVTVSGSESGDYK
DDDDKHHHHHH

(11) HER2Nb-sfGFP-H_6

MGQVQLQESGGGSVQAGGSLKLTCAASGYIFNSCGMGW**Y**RQSPGRERELVSRISGDGDTWHKESVKGRFTISQDN
VKKTLYLQMNSLKPEDTAVYFCAVCYNLETYWGQGTQVTVSSGGGSGGGSASSKGEELFTGVVPILVELDGDVNG
HKFSVRGEGEGDATNGKLTLKFICTTGKLPVPWPTLVTTLTYGVQCFSRYPDHMKRHDFFKSAMPEGYVQERTISF
KDDGTYKTRAEVKFEGDTLVNRIELKGIDFKEDGNILGHKLEYNFNSHNVYITADKQKNGIKANFKIRHNVEDGSV
QLADHYQQNTPIGDGPVLLPDNHYLSTQSVLSKDPNEKRDHMVLLEFVTAAGITHGGSESGDYKDDDDKHHHHHH

(12) GFP(minimizer)Nb-H_6

MGADVQLQESGGGSVQAGGSLRLSCAASGDTFSSYSMAWFRQAPGKECELVSNILRDGTTTYAGSVKGRFTISRDD
AKNTVYLQMVNLKSEDTARYYCAADSGTQLGYVGAVGLSCLD**Y**VMDYWGKGTQVTVSGSESGDYKDDDDKHH
HHHH

SUPPORTING REFERENCES

[1] K. R. Schmitz, A. Bagchi, R. C. Roovers, P. M. van Bergen en Henegouwen, K. M. Ferguson, *Structure* **2013**, *21*, 1214-1224.

[2] M. D'Huyvetter, J. De Vos, C. Xavier, M. Pruszynski, Y. G. J. Sterckx, S. Massa, G. Raes, V. Caveliers, M. R. Zalutsky, T. Lahoutte, N. Devoogdt, *Clin Cancer Res* **2017**, *23*, 6616-6628.

[3] A. Kirchhofer, J. Helma, K. Schmidthals, C. Frauer, S. Cui, A. Karcher, M. Pellis, S. Muyldermans, C. S. Casas-Delucchi, M. C. Cardoso, H. Leonhardt, K. P. Hopfner, U. Rothbauer, *Nat Struct Mol Biol* **2010**, *17*, 133-138.

[4] J. K. Böcker, W. Dörner, H. D. Mootz, *Chem Commun (Camb)* **2019**.

[5] S. Palei, K. S. Becher, C. Nienberg, J. Jose, H. D. Mootz, *Chembiochem* **2019**, *20*, 72-77.
